# The Conservative Treatment of Enlarged Prostate*Stenographically reported for this Journal by C. C. Mapes, Louisville, Ky.

**Published:** 1898-01

**Authors:** A. Morgan Vance

**Affiliations:** Louisville, Ky.


### Southern Medical Record.

A MONTHLY JOURNAL OF MEDICINE AND SURGERY.

VOL. XXVIII. ATLANTA, GA., JAN. 1898. NO. 1

### Original Articles.

### THE CONSERVATIVE TREATMENT OF ENLARGED PROSTATE.*

By A. MORGAN VANCE, M. D., Louisville, Ky.

In a paper bearing the above title,† read before the Louisville Surgical Society, the author said, in part:

I will discuss neither the etiology nor the pathology, but simply the treatment.

The history we receive when called to a man suffering from an enlarged prostate is nearly always the same: He has been compelled to get up at night frequently to relieve his bladder, and after exposure, or circumstances beyond his control, has been unable to void his urine, the bladder is distended, when he finds urination impossible. His age may be anywhere between forty and a hundred years when this first difficulty arises. Often at this first call a fatal mistake is made by the medical attendant, in the use of a catheter and the prescription for one to be used by the patient. The average catheter, if a soft one, is taken from the inside vest pocket, where possibly it has rested for days or weeks since it was last called into service. If thoroughly washed, it would not recognize itself. Unfortunately, this is rarely done, but on top of its already foul surface is smeared some rancid lard, or other lubricant furnished by the family, and into the bladder it goes. The result is easily imagined. If the attendant carries a pocket-case, a still more diabolical machine is forthcoming—an old, jointed, silver catheter that could not possibly be cleansed—and

*Stenographically reported for this Journal by C. C. Mapes, Louisville, Ky.

†This paper in the original appeared in the *Medical Times*, New York; the discussion which follows was omitted.—C. C. M.

much more damage is done from rough usage. A catheter can not be rendered aseptic by the ordinary patient, and should be used only by one thoroughly acquainted with its dangers, and by him as little as possible, even then. This little instrument kills more old men, and makes imperative the call for grave surgical interference, many times oftener than does the simple enlargement of the gland. It is the first step, in the majority of cases, towards a consultation with the undertaker.

The proper management of this condition depends almost entirely on the education of the patient in matters of prevention. He must learn how to avoid a repetition of the retention, and so insure a complete emptying of the bladder at each micturition.

If there is no organic stricture, in ninety-nine chances in one hundred the bladder can be emptied under the relaxing influences of a hot hip-bath, given in front of a fire in a very warm room. Should this fail, put the patient in bed, wrapped in warm blankets, and carefully pass a series of solid sounds well lubricated with sterilized oil; at the same time give quinine, ten grains, with phenacetine, five grains; wait half an hour and try the hip-bath again. I might be asked, Why not use a catheter instead of a sound? Because the weight of the sound enables you to enter the bladder with less difficulty and less danger; it dilates the canal through the congested prostate, and if the urine is not then voided voluntarily a catheter will follow the sound with less difficulty than if used primarily. Advise the patient to remain in bed, empty the bowels by an enema, if this has not been done before the bath, castor oil to follow, and the next urination will be accomplished with little difficulty. Before discharging the patient, give him full instructions as to his future actions. He should dress carefully, wearing warm flannel underwear, warm, thick footwear, heavy, thick shoes, as, of all the causes of trouble, cold is the greatest. His urinating must be done in a warm room, and not in the yard, clad only in his shirt. His bowels should be kept regulated, and he should be careful in his eating. Alcohol in moderation does good for almost all old men.

He should, by the use of abundant pure water, either carbonated or not, keep the urine pure. Boric acid is the sheet-anchor as a medicament for this purpose, salol coming next; strychnine may be given if there is need of general muscular tone. Opium should never be administered to a man with an enlarged prostate. Often have I known old men to be started rapidly on the downward path by a hypodermic injection of morphine given to relieve pain due to some accidental injury. Opium destroys what little power there is to empty the bladder, necessitating the catheter, and the usual consequences. Advice against old men marrying young women. One old fellow of seventy-six years, I remember, said: "Doctor, whenever

I have anything to do with a girl, blood passes with my urine." Following this idea, there should be absolute avoidance of all aphrodisiac remedies.

Disturbances at night can be reduced by withholding fluids late in the day, but at midnight, if awake for the purpose of emptying the bladder, a cracker with a glass of milk will often help to gain sleep the remainder of the night. Old men get hungry and need food oftener than the ordinary three times a day.

Any intercurrent troubles, such as oxaluria, or the uric acid habit, should be carefully attended to and proper attention paid to the functions of the skin.

If these rules be followed, the majority of these cases will live with fair comfort, and thus avoid the catheter, which, I am sure, is the cause of nearly all the conditions which call for the now too prevalent surgical procedures of cystotomy, castration, the ligature operation and the removal of the gland. Old men do not bear surgery well, and the miserable conditions which make them submit to almost anything are due not to the enlarged gland, but to the tinkering with a viscus, which tolerates any entrance from without very poorly.

The common practice of irrigation is to be condemned; use the kidneys as an irrigator, and better results will come. As a rule, the necessity for getting up at night can be reduced to one time after retiring. I have carried many old men up into the eighties without using the catheter, having been first called to relieve retention. I would almost rather give relief by the aspirator than the catheter, so convinced am I of the banefulness of its use. This may sound simple in the face of recent work in the way of radical treatment of enlarged prostate, but we know our proneness to take up new fads in surgery, as well as in other things. After watching the reports *pro* and *con*, I am inclined to doubt whether there is any good effect other than that resulting from the increased care given these old patients after the operation of castration or use of the ligature. There is no question that the mental effect is often distressing. The operation of removing the gland is one of great difficulty and possessed of considerable danger, and few men will submit to it. Drainage by cystotomy is all right for the relief of cystitis due to infection from without. When cystitis is not present, or will yield to rest and proper treatment through the kidneys, I think it never justifiable. You can always dilate and keep dilated the prostatic urethra. I will mention the case of an old man, aged eighty-two years, in which I was the consultant, where a nasty cystitis was present—due, evidently from the history, to a foul catheter—where the epicystotomy was made very easy by simply cutting down on the point of a large, hollow staff, fully a No. 36 size, which was passed into the bladder without difficulty.

The vast majority of men who reach the so-called catheter age live out their natural lives without the aid of physician or surgeon. It is admitted that nearly all old men have prostatic hypertrophy, but only a small per cent. have the accidents mentioned, and have to run the gauntlet of the catheter brigade with the risk of having added to their bearable trouble chronic cystitis, urethral fever, perineal abscess, etc., etc. Try conservative management of old men patients, and those of you who are prone to operate often will change your minds.

DISCUSSION.

DR. LOUIS FRANK: Conservative treatment is inapplicable where enlargement involves the middle lobe of the prostate. Such men should be instructed to pass their urine frequently, not allowing the bladder to become full. Where possible the use of the catheter should be avoided, particularly because of the liability to infection. There is little danger of decomposition of residual urine so long as there is no contamination from the atmosphere, even if the condition causing residual urine has extended over quite a period of time, but the bladder should not be subjected to catheterization and thus become accustomed to artificial evacuation; the muscular fibres should be allowed to exert their own force. This is true of the bladder under all conditions. A patient whose bladder has been subjected to distention for some time, becomes unable to pass his urine. In prostatic troubles the use of the catheter brings about the same condition. If there is distention of the bladder, repeated catheterization only makes the matter worse.

I believe dilatation, carried out properly with sounds, keeping the canal well opened, where there is enlargement of the middle lobe, passing the urine before the bladder becomes full and distended, is the better plan of treatment.

I know many old men who have no prostatic enlargement. Most old men use more or less alcoholic stimulants, and probably do not take care of themselves as they should. The use of the catheter is merely a habit in many cases, the bladder becomes slightly irritable and every time they desire to urinate they use the catheter, whether it is necessary or not.

While we do not look for prostatic enlargement in young men, even where they practice excessive venery, yet they sometimes have prostatic trouble. I believe these cases do not require the use of the catheter. With proper treatment by local applications of heat, etc., reducing the congestion caused by prolonged erections and excessive intercourse, the trouble will disappear.

As to castration for enlarged prostate: In some cases there may be a decrease in the size of the gland following this operation, but I question whether it is sufficient to relieve the trouble. Much of the good resulting from the operation is due to the mental impression made upon the patient. Moreover, they probably take better care of themselves after they have had trouble with the bladder, and much of the improvement is due to this fact. The results of castration for enlarged prostate, we will find when we have seen more of these cases will be about the same as in removal of the ovaries for the cure of myomatous tumors of the uterus, though I do not believe the conditions perfectly analogous.

DR. W. L. RODMAN: I have listened to the paper with interest, and in main agree in the conclusions stated. In the majority of cases, however, we will not see the patient in the first attack of retention, and will find the bladder already septic, probably from the use of an infected catheter, and in such cases the best treatment is not only to flush the bladder from behind, by large quantities of water, but to give boric acid internally to sterilize the urine. I believe that boric acid should be used locally as well as constitutionally.

DR. H. H. GRANT: I did not understand the author to clearly indicate that there is at the beginning an acute attack which introduces all these cases; and if we could see the patient in the acute attack, which is a simple congestion of the prostate gland, an inflammation caused by a slight cold, perhaps, and then employ the method of treatment he has suggested, we would probably often obtain relief, in simpler cases where the enlarged prostate is the first cause of the trouble. Many cases get well without the use of the catheter during the acute attack, but the hypertrophy continues, and in a considerable proportion of cases that have come under my observation, there has been no other treatment instituted except such as Dr. Vance has suggested. But at this time the bladder has been greatly distended by long continued residual urine, until it has in part lost its control, its power of contractility is greatly diminished, and no treatment can be looked upon as hopeful of recovery as long as this tendency to. retention of urine goes unrelieved.

Some cases are not seen until they are in bed with acute diseases. Three cases which have come under my observation in the last two years, in old men, had no difficulty in relieving the bladder until they were attacked by acute disease, two of them having what is commonly known as the "grip," the other having pneumonia. In both cases of "grip" the catheter was introduced at once, a step made necessary by the condition of the patient, and a large amount of residual urine was withdrawn. The bladder was found not only to be greatly distended but was capable of holding much more urine than its normal capacity. A condition of this kind occurring in an acute disease naturally requires the use of the catheter. As to the introduction of sounds under these circumstances, I did not employ this measure and question whether it would have accomplished anything, but I introduced in each of these three cases a catheter with a large curve at its distal extremity, the catheter being in one piece, made especially for the purpose, and no trouble attended its introduction. In none of these three cases did I discover that there was any particular trouble resulting from the introduction of the catheter. The patient who had pneumonia died; the other two with grip recovered and one of them was put upon the habitual use of the catheter without any disadvantage. The other one entirely recovered and was free from special trouble of any kind, though I learn he was subsequently attacked with a similar condition and died. During the last attack he was under the care of another physician who had previously attended him, but who was absent from the city when I was called in the first attack. Whether a catheter was used in the second attack or not, I do not know, but I do know that he had a great deal of difficulty with his bladder when he suffered from the grip at the time I attended him, and that he died in a subsequent attack. I judge, from what I have since heard of the case, that about the same line of treatment was carried out during the fatal attack that I had employed previously.

These cases constitute two classes, one in which there has been no treatment of the patient until the bladder is greatly dilated and contains a large amount of residual urine, which perhaps is not septic; and the proposition that Dr. Frank makes is not an unfair one, viz.: that sepsis is not likely to take place in residual urine until infection is introduced from without. What injures the bladder more than this, however, is that poisonous ingredients contained in residual urine are absorbed, and where the patient retains urine in this manner, which may be for weeks or months, which gradually becomes poisonous, by constant absorption of the poisonous ingredients, he is not only unfit for any operative measures, but is unfit to resist even simple catheterization.

In many cases which have not come properly under the care of the careful surgeon, the constitution has been undermined by long retention of urine and the absorption of poison, the patient dies, not so much from the effect of the catheter as from the fact that he is unable to resist disease, and then after simple withdrawal of the urine the man sinks into a typhoid state and dies, the death being attributed to the introduction of a catheter, when the real cause of death is the absorption of poison from the urine. It would be better, of course, if the patient could be relieved without the use of the catheter, but most of the cases to which I have been called and in which I have used the catheter, have been of the class I have just described.

The other class in which the trouble first makes its appearance as an acute condition, can be relieved in no other way except by prompt catheterization. I have in some cases employed irrigation with boric acid, but the cases in which I have had occasion to do much treatment of the bladder have been very grave and most of them have been unsatisfactory.

DR. A. M. VANCE: I do not claim that conservative treatment will have good effect in aggravated cases. I referred to the walking cases or the typical cases we see at first. In such cases I believe we are all agreed that the conservative treatment is indicated.

If there is a pocket behind an enlarged prostate, unless you put the patient on his belly during catheterization, there will still be inability to get the residual urine.

I believe the main thing is to get these men in good condition generally, then keep the urine pure, and when you have obtained relief by relaxation from hot baths as I have suggested, improve their general condition by tonics, keep them as near par as possible in the way of general health, and I feel sure if this is done and the patients instructed to exercise proper care in these matters, that we will have much less cystitis.

That the urine of old men is more or less ammoniacal has been conceded, and retention has occurred from simple cold. We should teach these patients how to prevent the complications to which I endeavored to call attention in my paper, and I am sure it can be done in the vast majority of cases.

